# Treatment with retinoic acid and lens epithelial cell-conditioned medium *in vitro* directed the differentiation of pluripotent stem cells towards corneal endothelial cell-like cells

**DOI:** 10.3892/etm.2014.2103

**Published:** 2014-12-03

**Authors:** PING CHEN, JUN-ZHAO CHEN, CHUN-YI SHAO, CHUAN-YIN LI, YI-DAN ZHANG, WEN-JUAN LU, YAO FU, PING GU, XIANQUN FAN

**Affiliations:** 1 Department of Ophthalmology, Ninth People’s Hospital, Shanghai Jiaotong University School of Medicine, Shanghai 200011, P.R. China; 2 Department of Ophthalmology, Renji Hospital, Shanghai Jiaotong University School of Medicine, Shanghai 200011, P.R. China

**Keywords:** embryonic stem cell, induced pluripotent stem cell, corneal endothelium cell, retinoic acid, conditioned medium, differentiation

## Abstract

Embryonic stem cells (ESCs) and induced pluripotent stem cells (iPSCs) have extensive self-renewal capacity and the potential to differentiate into all tissue-specific cell lineages, including corneal endothelial cells (CECs). They are a promising prospect for the future of regenerative medicine. The method of derivation of CECs from ESCs and iPSCs, however, remains to be elucidated. In this study, mouse ESCs and iPSCs were induced to differentiate into CECs using CEC embryonic development events as a guide. All-*trans* retinoic acid (RA) treatment during the embryoid body (EB) differentiation step was used to promote neural crest (NC) cell differentiation as first step and was followed by a second induction in CEC- or lens epithelial cell (LEC)-conditioned medium (CM) to ultimately generate CEC-like cells. During the corresponding differentiation stages, NC developmental markers and CEC differentiation markers were detected at the protein level using immunocytochemistry (ICC) and at the mRNA level by reverse transcription-quantitative polymerase chain reaction (RT-qPCR). During the first stage, the data indicated that 4 days of treatment with 1 μM RA starting on day 4 of EB formation favored NC cell differentiation and that plating on gelatin-coated plates led to cell migration out of the EBs. The second-stage differentiation results showed that the CM, particularly the LEC-CM, enhanced the yield of polygonal cells with CEC-specific marker expression shown by ICC and RT-qPCR. This study demonstrates that mouse ESCs and iPSCs were induced and expressed CEC differentiation markers when subjected to a two-step inducement process, suggesting that they are a promising resource for corneal endothelium failure replacement therapy in the future.

## Introduction

Corneal endothelium failure resulting from a reduction in cell number or cellular dysfunction leads to blindness that is treatable by the replacement of functional corneal endothelial cells (CECs); however, this treatment is hampered by the fact that human CECs have a poor or absent self-renewal capacity (1). Pluripotent stem cells (PSCs), such as embryonic stem cells (ESCs) and induced pluripotent stem cells (iPSCs), have gained widespread attention for their hallmark properties of extensive self-renewal capacity and developmental pluripotency for all cell lineages under the appropriate conditions (2,3). iPSCs hold great promise for replacement therapies in regenerative medicine and the modeling of numerous diseases that are currently unresponsive to traditional clinical approaches because the generation of patient-specific iPSCs directly from somatic cells renders the use of oocytes or embryos unnecessary (4–7). Pluripotent cells represent a powerful tool for tissue regeneration; however, until now, no protocol has been established for the directed differentiation of PSCs into CECs *in vitro*. This is likely due to a lack of knowledge about the molecular mechanisms by which ESCs develop into CECs. Development studies have revealed that CECs originate from differentiation of the first cranial neural crest (NC) cell wave during vertebrate embryo development (8). The NC is a transient structure in vertebrate embryos that initially generates NC stem cells, which then migrate throughout the body to produce a diverse spectrum of differentiated tissue types, including CECs (9). Therefore, NC derivatives from iPSCs have been considered for use in the first stage of the induction of CEC differentiation. However, to the best of our knowledge, the differentiation of NC stem cells after they reach their target site, which is located beneath the primary stromal layer synthesized by the corneal epithelium and adjacent to the lens epithelial layer, has not been described. Knowledge of the mechanisms governing NC cell differentiation to CECs remains rudimentary. It is accepted that the microenvironment, or niche, surrounding migrating cells, for example, lens epithelial cells (LECs), determines their ultimate fate during differentiation (10). However, the understanding of CEC differentiation remains limited by the lack of defined components that can be used as inducers of differentiation. The aim of the present study was to evaluate the hypothesis that reproducing the assembly of the CEC niche using a microenvironment containing LECs and CECs may induce differentiation. Therefore, co-culture with these potentially differentiation-inducing cells was used during the second differentiation step to further guide PSC-derived NC cells to the CEC fate.

## Materials and methods

### Preparation of differentiation-inducing cells

A total of 20 New Zealand White rabbits and 40 C57BL/6J mice were obtained from the Shanghai Animal Experimental Center (Shanghai, China), and all procedures were approved by the Animal Research Committee of the Ninth People’s Hospital, Shanghai Jiaotong University School of Medicine (Shanghai, China). Rabbit CECs and LECs were isolated from rabbit eyes and cultured in fresh growth medium (GM), which was Dulbecco’s modified Eagle’s medium (MEM)/F12 (Invitrogen Life Technologies, Carlsbad, CA, USA) medium containing 10% fetal bovine serum (FBS; Invitrogen Life Technologies) and 100 U/ml penicillin-streptomycin (Invitrogen Life Technologies). After eyeball enucleation and corneal dissection, the intact Descemet’s membrane and lens anterior capsule tissues were removed with micro-forceps and incubated in GM at 37°C overnight. Tissues were then digested with 0.25% trypsin and 0.02% EDTA (Sigma-Aldrich, St. Louis, MO, USA) to acquire a single-cell suspension. After the cells were passed through a 100-μm mesh strainer, they were centrifuged at 200 × g for 4 mins and subsequently seeded in T25 flasks at a density of 2×10^5^ cells/ml. When the cells reached 80% confluence after the second passage, the GM was replaced with CEC defined medium (CEC-DM), which was DMEM/F12 medium containing 20 ng/ml basic fibroblast growth factor (recombinant murine bFGF; Invitrogen Life Technologies), 1% N2 neural supplement (Invitrogen Life Technologies), 2% B27 supplement (Invitrogen Life Technologies), 50 μg/ml ascorbic acid (Roche, Bromma, Sweden) and 100 U/ml penicillin-streptomycin. After 12 h of culture, the conditioned medium (CM) was collected and filtered using a 0.22-μm filter unit and stored at −80°C for subsequent co-culture.

### Culture of mouse ESCs and iPSCs

The mouse ESC line R1, a gift from Professor Yilin Cao (Key Laboratory of Tissue Engineering, National Tissue Engineering Center of China, Shanghai, China), and iPSCs derived from mouse neural progenitor cells, a gift from Professor Jin Yin (Key Laboratory of Stem Cell Biology, Institute of Health Sciences, Shanghai Institutes for Biological Sciences, Chinese Academy of Sciences, Shanghai, China), have been involved in previous studies (1,11). Early-passage PSCs (less than P8) were used in the present study. Mouse embryonic fibroblasts (MEFs) were derived from mouse embryos at embryonic day 13.5, and early-passage cells (P2–P3) were used as feeders for the culture of ESCs and iPSCs. To maintain their undifferentiated state, R1 ESCs and iPSCs were plated onto a feeder layer of MEFs treated with 10 μg/ml mitomycin-C (MMC) for 2 h, seeded onto T25 flasks pre-coated with 0.1% gelatin and grown in embryonic stem medium (ESM), which was Iscove’s modified Dulbecco’s medium (IMDM) supplemented with 15% FBS, 2 mM L-glutamine, 1% non-essential amino acids (NEAA), 1 mM pyruvate, 0.1 mM β-mercaptoethanol (all from Invitrogen Life Technologies) and 1,000 U/ml leukemia inhibitory factor (LIF; Millipore).

### Step one: NC differentiation after EB formation with RA treatment

To reduce the influence of the feeder layer on differentiation, mouse R1 ESCs and iPSCs were trypsinized, dissociated into single-cell suspensions and subcultured onto 10-mm dishes pre-coated with 0.1% gelatin. After two days of culture in ESM, the cells were trypsinized, centrifuged at 200 × g for 4 mins and resuspended. The supernatant containing predominantly ESCs or iPSCs was collected and resuspended in EB differentiation medium (EBM) without LIF at a density of 1.0×10^5^ cells/ml. Low-adherence dishes were used to facilitate the suspension culture. The medium was changed every 2 to 3 days after collecting EBs by gravity sedimentation. All-*trans* retinoic acid (RA; Roche, Sweden) was added to the EBM on day 4 of EB differentiation at various final concentrations (0.1, 0.5, 1 or 10 μM), and the cells were cultured for an additional 4 days (EBd4+4). The cells were collected for total RNA extraction at multiple time points during EB differentiation: on day 4 (EBd4) and after the addition of RA, recorded as EBd4+n, where n = the number of RA exposure days (for example, EBd4+2, EBd4+4 and EBd4+6). The mRNA expression profile was obtained using reverse transcription-quantitative polymerase chain reaction (RT-qPCR). Additionally, EBs were seeded on gelatin-coated glass coverslips (VWR, West Chester, PA, USA) on day EBd4+3 through EBd4+4 to allow cells to adhere and migrate, and then fixed for analysis by immunocytochemistry (ICC).

### Step two: Induced NC differentiation towards CEC-like cells

#### Effects of different coatings on EB migration and differentiation

To optimize the induction conditions, different plate coatings were compared, as the coating serves as an extracellular matrix (ECM) component and plays an important role in cell differentiation. Gelatin, fibronectin (Fn) and laminin (Ln) were assayed. The Fn and Ln (at 2 μg/cm^2^) coatings were kept at 37°C for 2 h before use. The gelatin coating was incubated at 37°C for >24 h. After treatment with 1 μM RA, EBs were plated on various coatings and switched to LEC-CM to gain insight into the impact of ECM on induced CEC differentiation.

#### Cell differentiation in different co-culture environments

For the next stage of differentiation, following the treatment with 1 μM RA on day 4 (EBd4+4), EBs were transferred to a co-culture environment; various co-culture systems were compared. EBs were first collected by gravity sedimentation for 10 min, after which the supernatant was removed. After being washed in serum-free DMEM/F12, EBs were plated on gelatin-coated 6- or 12-well plates in one of the following media to induce CEC-like cell differentiation: CEC-CM, LEC-CM and CEC-DM. The cells were cultured at 37°C in a 5% CO_2_ humidified atmosphere. During the 7–10-day co-culture experiment, media were changed every 2 to 3 days. At the end of the induction, the cores of the endothelium-like colonies were examined under a microscope (M620, Leica, Wetzlar, Germany) and removed using needles to ensure that the majority of the endothelium-like population was harvested for RNA extraction and analysis.

#### RT-qPCR

Total RNA was extracted from the cultured cells using TRIzol reagent (Invitrogen Life Technologies), and first-strand cDNA synthesis was performed using the PrimeScript™ RT reagent kit (Perfect Real Time; Takara, Dalian, China) (12). qPCR was performed in a 20-μl reaction containing 10 μl reaction mixture, 1 μl cDNA, 2 μl primers (Table I) and 7 μl ddH_2_O. qPCR was conducted using a 7500 Real-time PCR Detection System (Applied Biosystems, Irvine, CA, USA) after a hot start at 95°C for 10 min and 40 cycles of amplification (15 sec at 95°C and 1 min at 60°C) (13). The efficiency of the reaction primers was measured using serial dilutions of the cDNA (1:1, 1:5, 1:25, 1:125, 1:625 and 1:3,125). Each sample was tested in triplicate. The relative gene expression was analyzed using the Pfaffl method (3). Data are expressed as a fold change relative to untreated controls after normalizing to the β-actin endogenous control.

#### ICC

Alkaline phosphatase (ALP) activity assay of PSCs was performed using an ALP staining kit (Sigma-Aldrich) according to the manufacturer’s instructions. Cells were seeded onto glass coverslips pre-coated with gelatin that had been placed in 12-well plates. At various differentiation time points, they were fixed in 4% (w/v) paraformaldehyde (Sigma-Aldrich) in 1× phosphate-buffered saline (PBS; 2.68 mM KCl, 1.47 mM KH_2_PO_4_, 135.60 mM NaCl and 8.10 mM Na_2_HPO_4_) for 15 min at room temperature. The cells were then washed in PBS prior to incubation with antibody blocking buffer [PBS containing 10% (v/v) normal goat serum (Invitrogen Life Technologies), 0.3% TritonX-100 (Sigma-Aldrich) and 0.1% NaN_3_ (Sigma-Aldrich)] for 1 h at room temperature. The incubation with primary antibodies (Table II) was performed overnight at 4°C. After three washes for 5 min in PBS, the coverslips were incubated with fluorescently labeled secondary antibodies (Alexa Fluor 546 or FITC 488 goat anti-mouse or goat anti-rabbit IgG, 1:800 in PBS; Invitrogen Life Technologies) for 1 h at room temperature. After washing in PBS, the cell nuclei were counterstained with 4′,6-diamidino-2-phenylindole (DAPI; Invitrogen Life Technologies) for 5 min at room temperature. Negative control samples were processed in parallel in the absence of primary antibody. Immunoreactive cells were visualized and photographed using a fluorescence microscope (Olympus BX51, Japan).

#### Statistical analysis

The experimental statistics presented in this study are expressed as the mean ± standard derivation (SD). All experiments were performed ≥3 times unless otherwise specified. Data were analyzed using Student’s two-tailed t-test to compare the means of two groups or a one-way analysis of variance (ANOVA) for comparison of the means of more than two groups using SPSS Statistics version 17.0 software (SPSS, Inc., Chicago, IL, USA). P<0.05 was considered to indicate a statistically significant difference.

## Results

### Culturing differentiation-inducing cells (CECs and LECs) for the co-culture system

**Figure 1:**
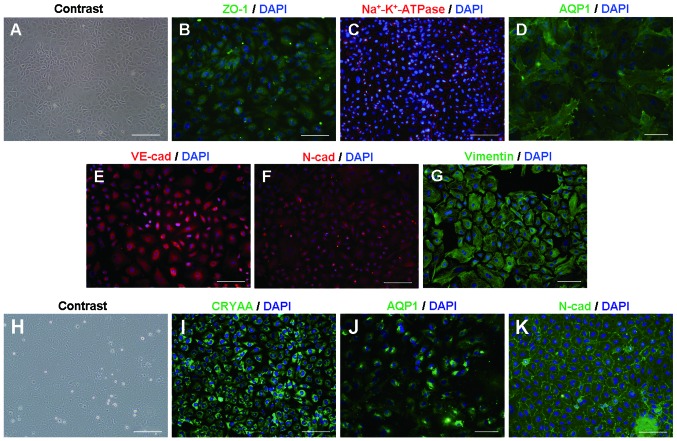
Characterization of the CEC and LEC *in vitro* cultures. (A) *In vitro* cultured CECs from rabbit presented as a hexagonal or polygonal monolayer and were arranged in a cobblestone pattern that resembles the structure of this tissue *in vivo*. When specific markers were analyzed by immunocytochemistry, CECs stained positive for the expression of (B) the tight junction marker ZO-1, (C) Na^+^-K^+^-ATPase α-subunit, (D) AQP1, (E) VE-cadherin, (F) N-cadherin and (G) vimentin. All nuclei were counterstained with DAPI. Membrane localization was observed for Na^+^-K^+^-ATPase, N-cadherin and AQP1. Cytoplasmic localization was observed for vimentin and VE-cadherin. ZO-1 was not well confined to the membrane. (H) The phenotype of the LECs was similar to that of CECs with a polygonal monolayer, and showed (I) high crystallin-α A (CRYAA) cytoplasmic expression and (J) the expression of AQP1 and (K) N-cadherin at the membrane. Scale bars: 100 μm in A, C and H; 50 μm in B, E, F, I and K; and 20 μm in D, G and J. CEC, corneal endothelial cell; LEC, lens epithelial cell; DAPI, 4′,6-diamidino-2-phenylindole.

Rabbit CECs showed quick adherence and a positive growth state; these cells reached confluence at approximately the third day after the digestion from Descemet’s membrane and displayed hexagonal or polygonal morphology (Fig. 1A). Positive immunostaining of ZO-1, AQP1, Na^+^-K^+^-ATPase, VE-cadherin, N-cadherin and vimentin, which have been used as general markers for CECs, was used to identify the cultured cells as CECs (Fig. 1B–G).

Rabbit LECs adhered slowly to the plates and reached confluence on approximately day 7 of culture (Fig. 1H). Positive identification of LECs was made using immunostaining of LEC-specific crystallin-α A (CRYAA) and two associated proteins, AQP1 and N-cadherin (Fig. 1I–K).

### Analysis of iPSC and ESC culture and differentiation based on EB formation

**Figure 2:**
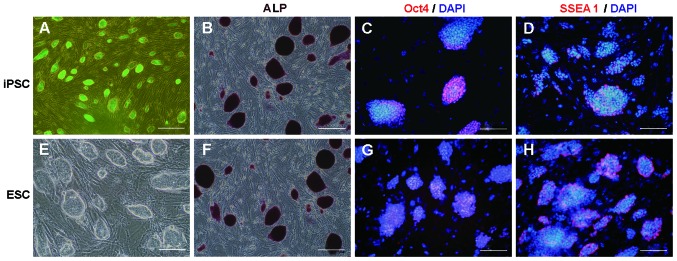
Pluripotency and the undifferentiated state of mouse iPSCs and ESCs. Mouse iPSCs and ESCs maintained their pluripotency and undifferentiated state in the presence of MEF-feeder cells and LIF. The PSCs developed into colonies. iPSCs expressed (A) EGFP, (B) alkaline phosphatase (ALP), (C) the pluripotent marker Oct4 (nuclear localization) and (D) the mouse-specific undifferentiated cell marker SSEA1 (membrane localization). (E) Mouse ESCs appeared similar to the PSCs in terms of colony morphology, and expressed (F) ALP, (G) Oct4 and (H) SSEA1. Scale bars: A, 100 μm, B-H, 50 μm. iPSC, induced pluripotent stem cell; ESC, embryonic stem cell; MEF, mice embryonic fibroblast; LIF, leukemia inhibitory factor.

Mouse iPSCs and ESCs were cultured in the undifferentiated state as well-shaped colonies on a feeder cell layer in ESM containing LIF. Surface antigen SSEA1, regarded as a marker of the undifferentiated state and ALP and Oct4, markers for pluripotency in the mouse, were detected by immunofluorescent staining and ALP staining (Fig. 2).

**Figure 3:**
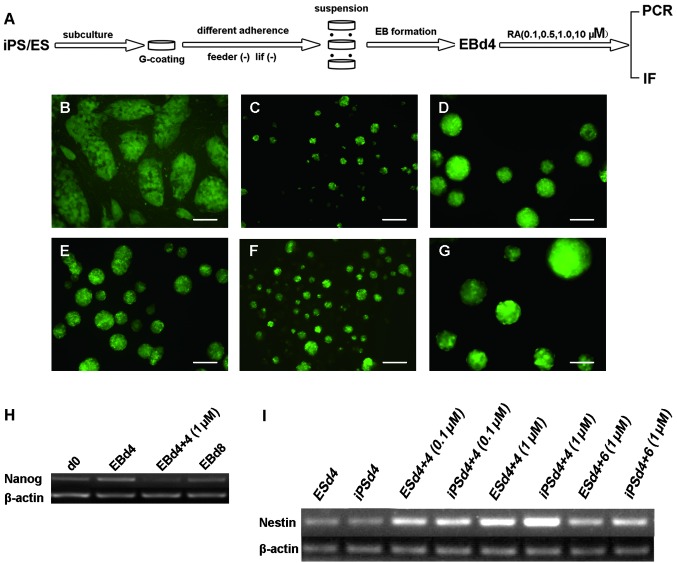
Neural crest differentiation of mouse pluripotent stem cells relies on EB formation. (A) The procedure for neural crest differentiation was based on EB formation and RA treatment. Microscopic views of iPS-EBs at various time points: (B) iPSCs subcultured on gelatin to reduce feeder cell numbers, (C) EBs formed 1 day after the removal of feeder cells and LIF, (D) EB spheres on EB differentiation day 4, (E) EB differentiation on day 4 (d4) and 1 μM RA treatment for another 4 days, (F) EB differentiation on day 4 and 10 μM RA treatment for another 4 days, and (G) EB differentiation on day 8 (d8) without RA treatment. RT-qPCR for (H) Nanog mRNA expression in iPS-EBs. and (I) Nestin mRNA expression in iPS-EBs and ES-EBs. Scale bars: A–F, 100 μm. EB, embryoid body; RA, retinoic acid; iPS, induced pluripotent stem; iPSC, induced pluripotent stem cell; ES, embryonic stem; LIF, leukemia inhibitory factor; RT-qPCR, reverse-quantitative transcription polymerase chain reaction. d4+n, day 4 plus the number of RA exposure days.

Fig. 3A demonstrates the procedure of PSC cell differentiation. Following the subculture of PSCs on gelatin-coated plastic plates with a reduced number of feeder cells for 2 days, the iPSC colony size was increased (Fig. 3B). The complete removal of LIF and feeder cells during suspension culture initiated the spontaneous differentiation of iPSCs into embryoid bodies (EBs; Fig. 3B–G). One day after the plating of iPSCs, the cells congregated and proliferated to form GFP-positive spheres, called induced pluripotent stem (iPS)-EBs (Fig. 3C). The spheres continued to grow until RA exposure on EBd4 (Fig. 3D). The addition of RA slowed the growth of the EB spheres. In the presence of 1 μM RA, EBs were no longer growing on EBd4+4 (Fig. 3E), in contrast to untreated EBs on EBd8 (Fig. 3G). Abundant apoptosis and cellular debris were observed at a higher concentration of RA (10 μM) at EBd4+4 (Fig. 3F).

### Effect of RA on the NC differentiation of iPSC- and ESC-derived EBs

The addition of RA accelerated the differentiation process, as shown by the severe reduction in the mRNA expression of Nanog, a marker of the undifferentiated state, at EBd4+4 after 1 μM RA treatment (Fig. 3H). The expression level of the neuroectoderm marker Nestin varied based on the duration and concentration of the RA treatment. The 1 μM RA treatment promoted peak Nestin mRNA expression at EBd4+4 for both iPS-EBs and ES-EBs (Fig. 3I). However, Nestin was downregulated at EBd4+6 after treatment with 1 μM RA, which indicated that neuroectoderm began to predominate in EBs at EBd4+4 following treatment with 1 μM RA in both types of PSCs.

**Figure 4:**
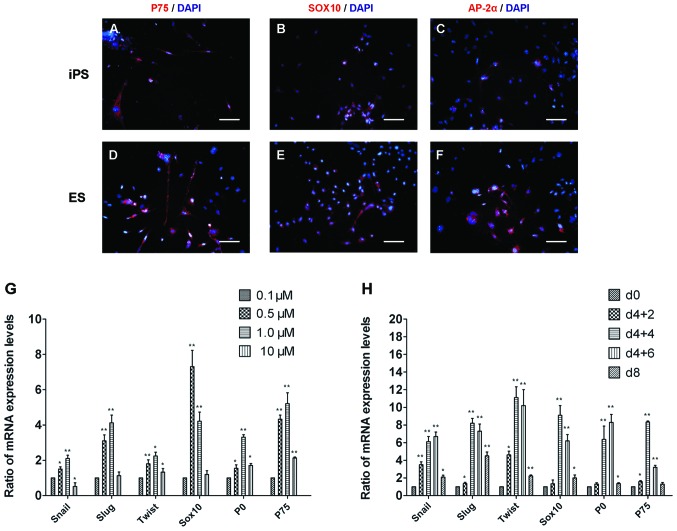
Characterization of neural crest differentiation in RA-induced EBs. The cells migrating from iPS-EBs were fixed and immunostained with antibodies against (A) P75, (B) SOX10 and (C) AP-2α and the results indicated that the cells had been induced toward neural crest differentiation. In ES-EBs, (D) P75, (E) SOX10 and (F) AP-2 α protein expression were also detected. Scale bars: A–F, 50 μm. (G) The gene expression profile of EBd4+4 days of RA treatment at various concentrations was determined by qPCR. (H) The mRNA expression levels of these genes compared with neural crest differentiation in 1 μM RA at different time points and undifferentiated iPSCs (day 0) cells were determined in the same way. qPCR data from the iPS-EBs. The values are expressed as means ± SEM (n=3). ^*^P<0.05, ^**^P<0.01 vs. the 0.1 μM or d0 group, in G and H, respectively. d0, iPSCs; d4+2, iPSCs after EB differentiation for 4 days plus 1 μM RA treatment for 2 days; d4+4, iPSCs after EB differentiation for 4 days plus 1 μM RA treatment for 4 days; d4+6, iPSCs after EB differentiation for 4 days plus 1 μM RA treatment for 6 days; d8, iPSCs after EB differentiation 8 days without RA treatment; RA, retinoic acid; EB, embryoid body; iPS, induced pluropotent stem; iPSC, induced pluripotent stem cell; ES, embryonic stem; qPCR, quantitative polymerase chain reaction.

NC marker protein expression was examined using ICC and gene expression was examined by RT-qPCR in the induced EBs. Soon after EBs were seeded onto gelatin-coated plates, cells migrated out of the EBs. The expression of the NC stem cell markers P75, SOX10 and AP-2α in the RA-induced EBs (Fig. 4A–F) was detected in the migrating cells around EB cores by ICC. The gene expression levels of Snail, Slug, Twist, Sox10, P0 and P75 were upregulated in the induced cells compared with the levels in undifferentiated cells. The effect of RA was concentration- and time course-dependent. Based on the gene expression profile of EBd4+4 EBs, RA at a concentration of 0.5–1 μM was better at promoting NC differentiation compared with other concentrations that were investigated (Fig. 4G). The mRNA expression of these genes was greatly upregulated in 0.5 μM and 1 μM RA but to a lesser extent in 10 μM RA; indeed, Snail was downregulated in 10 μM RA. All genes except SOX10, showed the highest mRNA expression level in 1 μM RA. The appropriate RA treatment time course was decided based on the mRNA expression profile analysis and the comparison of samples treated with 1 μM RA at the time points EBd4+2, EBd4+4, and EBd4+6 and samples without RA treatment at day 0 and day 8 (Fig. 4H). Most genes were induced to their relative highest expression levels at the EBd4+4 time point. Slug, Twist, Sox10, and P75 showed the highest level of expression at EBd4+4, while Snail and P0 showed higher levels of expression at EBd4+4 and their peak expression level at EBd4+6.

These data indicate that EB differentiation for 4 days followed by 1 μM RA treatment for another 4 days (EBd4+4, 1 μM RA) could be used to induce NC differentiation as the first step of CEC differentiation.

### Effect of ECM components on EB cell migration and differentiation

**Figure 5:**
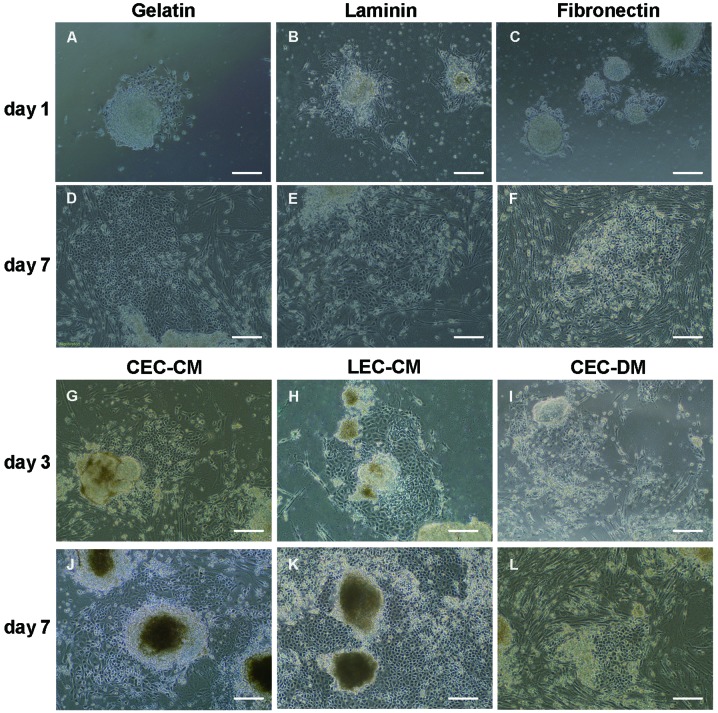
Morphology of EB cell migration and differentiation in CM. Diverse effects of different coatings on EB cell migration and differentiation were found. During early migration on day 1 and late migration on day 7 after EB growth on various coatings: (A, D) gelatin (B, E) laminin, and (C, F) fibronectin. EBs continued to differentiate on gelatin-coated plates in CEC-CM and LEC-CM with CEC-DM as a control. Cells migrated out of the EBs and became endothelium-rich colonies. Induced pluripotent stem cells (G) in CEC-CM, (H) LEC-CM and (I) CEC-DM on day 3; (J) CEC-CM, (K) LEC-CM and (L) CEC-DM on day 7. Scale bars: A–L, 100 μm. EB, embryoid body; CM, conditioned medium; DM, differentiation medium; CEC, corneal endothelial cell; LEC, lens epithelial cell.

The effects of the ECM components gelatin, Ln and Fn on EB cell migration and differentiation were investigated (Fig. 5A–F). During the first few days, cells on Ln (Fig. 5B) migrated faster, but the difference was small. On day 7, the superiority of gelatin in promoting cell migration (Fig. 5D) became clear. In comparison with Ln (Fig. 5E) and Fn (Fig. 5F), gelatin supported greater EB cell migration and formed stronger adherence of the endothelium-like colonies. Therefore, gelatin was used as the coating for cell culture and differentiation assays in subsequent experiments.

### Comparison of different co-culture systems for the second steps

Different co-culture models led to varied differentiation results observed in the center of the EBs. The CM induction cultures provided positive results (Fig. 5G–L); under these conditions, cells from the EBs migrated out of the colonies early, re-adhered to the gelatin coating, proliferated and differentiated into cells with a polygonal shape, resulting in endothelium-like colonies. Following cell migration and differentiation, classic-looking colonies appeared containing an endothelium surrounding the core of the EBs. Vigorous growth and significant endothelium differentiation, based on morphology, was enhanced in the CM, and particularly in the LEC-CM (Fig. 5H and K).

**Figure 6:**
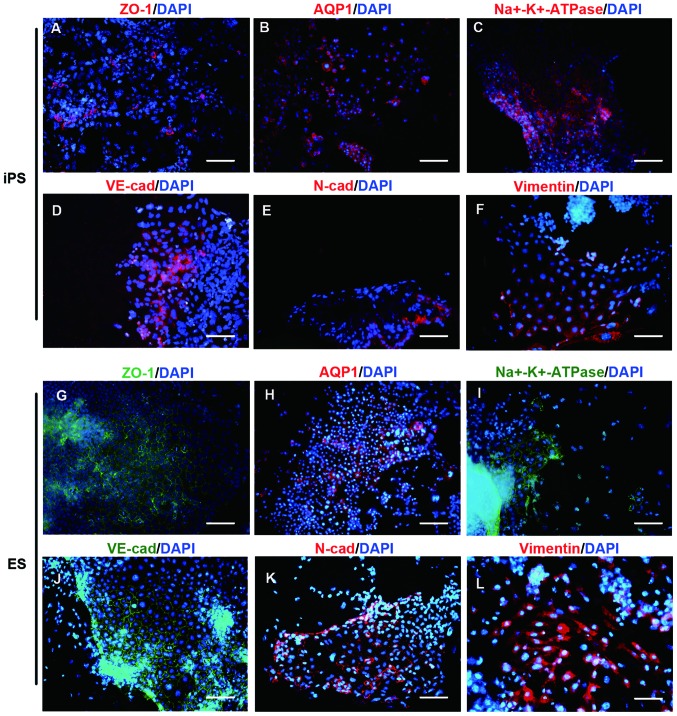
Protein expression of CEC-specific markers in CEC-like colonies induced by LEC-CM. During the second induction of differentiation by CM, endothelium-like colonies developed, and CEC-specific markers were shown to be expressed in the CEC-like populations by ICC after an LEC-CM induction of 7 days. Expression of (A) ZO-1, (B) AQP1, (C) Na^+^-K^+^-ATPase, (D) VE-cadherin, (E) N-cadherin and (F) vimentin was detected in iPSC derivatives. (G) ZO-1, (H) AQP1, (I) Na^+^-K^+^-ATPase, (J) VE-cadherin, (K) N-cadherin and (L) vimentin expression were similar in ESC derivatives. Scale bars: A, C, D, E, G, I, J and K, 50 μm; B and H, 100 μm; F and L, 20 μm. CEC, corneal endothelial cell; LEC, lens epithelial cell; CM, conditioned medium; ICC, immunocytochemistry; iPSC, induced pluripotent stem cell: ESC, embryonic stem cell.

The expression of common CEC marker proteins, including AQP1, ZO-1, Na^+^-K^+^-ATPase, N-cadherin, VE-cadherin and vimentin, was examined using ICC after LEC-CM induction for 7 days (Fig. 6). Positive expression of these markers was found in the polygonal cells of both origins. Certain markers were expressed differently between the pluripotent ES and iPS cell groups.

**Figure 7:**
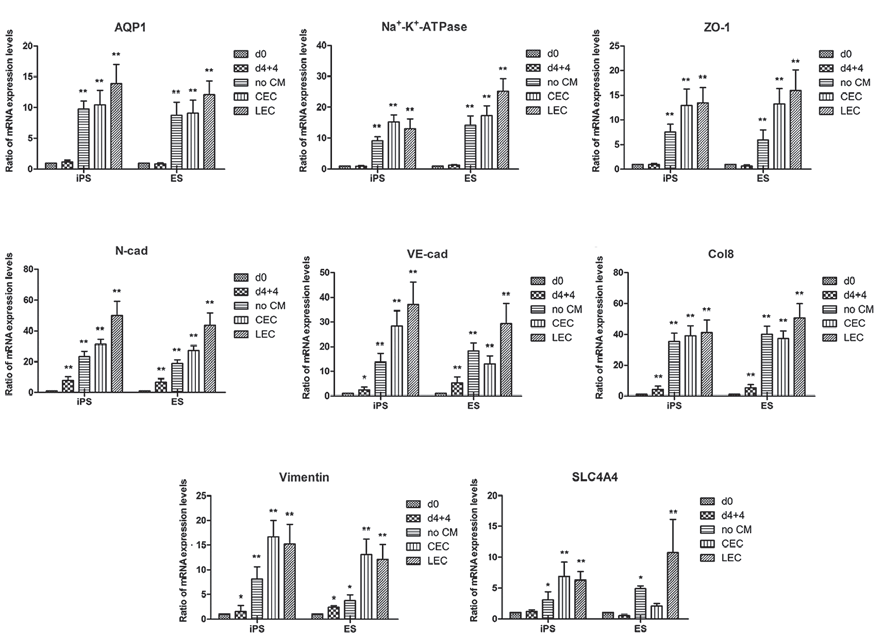
Induced mRNA expression profile of differentiation-specific genes in CEC-like cells. (A–H) Gene expression profiles of AQP1, Na^+^-K^+^-ATPase, ZO-1, N-cadherin (N-cad), VE-cad, Col8, vimentin and SLC4 A4 at day 0, during the first stage of differentiation, and during the second induction of differentiation using CEC-DM, CEC-CM or LEC-CM for 7 days. The error bars and statistical analysis show the standard error of the mean (n=3). The values presented are the mean ± SEM (n=3). ^*^P<0.05, ^**^P<0.01 compared with day 0. d0, undifferentiated iPSCs or ESCs, d4+4, EB differentiation for 4 days plus 1 μM RA treatment for 4 days; no CM, cells after the second induction of EBd4+4 in CEC-DM without CM for 7 days; CEC-CM, cells after the second induction of EBd4+4 in CEC-CM for 7 days; LEC-CM, cells after the second induction of EB d4+4 in LEC-CM for 7 days. CEC, corneal endothelial cells; DM, differentiation medium; CM, conditioned medium; LEC, lens epithelial cell; iPSC, induced pluripotent stem cell; ESC, embryonic stem cell; RA, retinoic acid; EB, embryoid body.

The gene expression levels of AQP1, ZO-1, Na^+^-K^+^-ATPase, N-cadherin, VE-cadherin, collagen VIII (Col8) and SLC4A4 in the differentiated cells were determined by RT-qPCR analysis. All marker genes were strongly upregulated in the differentiated cells compared with the undifferentiated cells (Fig. 7). At the mRNA level, in the iPSC derivatives and ESC derivatives, there were increases of 13.9- and 12.12-fold in AQP1; 13.42- and 15.92-fold in ZO-1; 13.12- and 25.1-fold in Na^+^-K^+^-ATPase; 15.2- and 12.1-fold in vimentin; 50.1- and 43.6-fold in N-cadherin; 37.1- and 29.3-fold in VE-cadherin; 41.22- and 50.73-fold in collagen VIII (Col8); and 7.36- and 10.09-fold in SLC4A4, respectively. Therefore, iPSCs and ESCs showed a similar potential for differentiation in this induction protocol, and LEC-CM was demonstrated to have the most efficient induction effect, as shown by the mRNA expression profile.

## Discussion

In this study, a protocol was developed for the induction of differentiation towards CECs from two types of mouse pluripotent stem cells (mPSCs): ESCs and iPSCs. The differentiation protocol is based on a two-step induction that begins with the formation of EBs and subsequent RA treatment followed by culture in CM. This co-culture model is sufficient to induce the mPSCs after ~17 days.

During the first stage of the protocol, EB formation, multiple cell lineages spontaneously differentiate but do not yield pure populations of cells, which complicates the characterization of the differentiated cells. RA is the active metabolite derived from the liposoluble vitamin A (retinol), which has extensive bioactivity and is increasingly recognized for its role in guiding embryonic development. It is well-established that exposure to RA for 4 days during embryoid body (EB) formation is associated with neural differentiation, as shown in previous studies (14–17). However, as this conventional approach is inefficient in inducing the differentiation of many different germ cell layers, including the ectoderm, mesoderm, endoderm and NC, it was modified in the present study to induce NC differentiation. The effective RA concentration was chosen based on its ability to upregulate the expression of epithelial-mesenchymal transition (EMT)-associated transcription factors and NC differentiation marker genes (18,19). In the present study, enrichment for NC cells by sorting or isolation after the first stage of the differentiation protocol was not carried out. Instead, the EBs were simply plated onto ECM. The ICC and other results confirmed that without trypsinization or sectioning, the NC stem cells residing in the EBs migrated out, survived and differentiated during the second stage of induction, which was treatment with CM and plating on gelatin-containing ECM.

Several lines of ESC differentiation research indicate that the ECM plays significant roles in cell proliferation, cell death, cell survival and differentiation (20,21); however, the role of the ECM in NC-to-CEC differentiation has not been studied in detail. Plate coatings such as gelatin, Fn and Ln, which are considered to be ECM in cell culture, were evaluated in the present study. When the EBs were grown on coated plates following RA exposure, differences were found in cell migration and cell morphology. The data showed that gelatin promoted cell migration and the generation of colonies with an endothelium-like appearance.

The co-culture method of differentiation, a simulation of cell-cell interaction, was used as a second induction step for derived NC cells. The mechanisms governing cell fate specification are not clear. LECs and CECs were selected as inductive cells for co-culture based on the understanding of the role of the lens epithelium in CEC organization during development. LECs can affect neighboring cells by a paracrine mechanism, although the signaling mechanisms are unclear. Mature CECs can also contribute to the niche by autocrine regulation and were used as an additional inducer. The findings of the present study are in agreement with previous reports (22,23), and demonstrate that CM from CECs and LECs can induce differentiation. Significant CEC-like differentiation was observed in the CM groups. CEC-CM and LEC-CM both enhanced CEC-like colony formation, as demonstrated by the polygonal cells and gene expression. CM from lens epithelium or corneal endothelium has been demonstrated to be beneficial for cell survival (24,25). Recent advances in generating functional corneal endothelium from corneal stromal stem cells of NC origin were found to depend on signaling by RA and Wnt/β-catenin (26). Whether the results in the present study were due to the same signaling pathway is currently under investigation.

To the best of our knowledge, this is the first report of the generation of CEC-like cells from mouse ESCs and iPSCs. As in most innovative work, clear morphological alterations were observed and a number of problems remained unresolved. Abundant polygonal-shaped cells that were arranged in cobblestone-like clusters in CEC-CM- and LEC-CM-treated plates were observed. The expression of CEC-specific proteins and mRNA was enhanced, including ZO-1, Na^+^-K^+^-ATPase, AQP1, N-cadherin, VE-cadherin, collagen VIII, vimentin and SLC4 A4 after inducement. However, the functions of the induced cells were not evaluated in this study. The main function of mature corneal endothelium is maintaining the stroma in a dehydrated state to support corneal transparency. Chamber and clinical observations made following transplantation in animal models of CEC failure are required as the next step, and will be the focus of future studies.

## Figures and Tables

**Table I:** Primers used for reverse transcription-quantitative polymerase chain reaction.

Genes	Accession no.	Forward primer	Reverse primer	Product size (bp)
Snail	NM_011427.2	ACACGCTGCCTTGTGTCTGC	TGGAGCAAGGACATGCGGGAGA	229
Slug	NM_011415.2	ACGCCTCCAAGAAGCCCAACT	TGGAGCTGCCGACGATGTCCATA	161
Twist	NM_011658.2	TTCAGACCCTCAAACTGGCGGC	ATCCTCCAGACGGAGAAGGCGT	139
Sox10	NM_011437.1	AGAAGGAACAGCAGGACGGCGA	TGACGTGCGGCTTGCTCTTG	150
P0	NM_008623.4	GGTGGTGCTGTTGCTGCTGT	GCAGCTTTGGTGCTTCGGCT	195
P75	NM_033217.3	AAAGCCTGCAACCTGGGCGA	TAGGAGCATCGGCACACGGCAT	200
Nestin	NM_016701.3	AACTGGCACACCTCAAGATGT	TCAAGGGTATTAGGCAAGGGG	235
β-actin	NM_007393.3	AGCCATGTACGTAGCCATCCA	CTCAGCTGTGGTGGTGAA	226
Nanog	NM_028016.2	TTGGTTGGTGTCTTGCTCTTT	CAGGAAGACCCACACTCATGT	196
AQP1	NM_007472.2	TGCTGGCGATTGACTACA	ACTGGTCCACACCTTCAT	200
ZO-1	NM_001163574.1	ACGAGGTTATTTCCAGCGTTT	GGTGGAACTTGCTCATAA	237
Na^+^-K^+^ -ATPase	NM_178405.3	ATCAGCGAGCTCAGGACATT	ACTACAGCCGCTAGCACGAT	226
Collagen VIII	NM_007739.2	CCATCACCCCAGGGAGAGTA	CCGGTGGGAAAGGTACAGTC	154
N-cadherin	NM_007664.4	CCTTCTGTGTATCATCATCCT	AGTCATAGTCCTGGTCTTCT	176
VE-cadherin	NM_009868.4	TGTGCTTGCCTATGAGAG	ACAGATGCGTTGAATACCT	177
Vimentin	NM_011701.4	TGGTTGACACCCACTCAAAA	GCTTTTGGGGTGTCAGTTGT	268
SLC4A4	NM_018760	GGTCACCACACGATCTACATTG	TTTGTCGGAGTAGTTCTCGGA	129

**Table II:** Primary antibodies used for immunocytochemistry.

Antibodies	Type	Source	Dilution
AQP1	Mouse monoclonal	Abcam	1:200
ZO-1	Rabbit polyclonal	Abcam	1:200
Na^+^-K^+^-ATPase	Mouse monoclonal	Abcam	1:200
N-cadherin	Rabbit monoclonal	Epitomics	1:200
VE-cadherin	Mouse monoclonal	Abcam	1:100
Vimentin	Rabbit monoclonal	Epitomics	1:200
Crystallin-αA	Rabbit monoclonal	Santa Cruz	1:100
Oct4	Mouse monoclonal	Millipore	1:200
SSEA1	Mouse monoclonal	Abcam	1:200
P75	Rabbit polyclonal	Abcam	1:300
SOX10	Rabbit polyclonal	Abcam	1:200
AP2α	Rabbit monoclonal	Epitomics	1:200

## Abbreviations

PSC: pluripotent stem cell
ESC: embryonic stem cell
iPSC: induced pluripotent stem cell
EB: embryoid body
LEC: lens epithelial cell
CEC: corneal endothelial cell
MEF: mouse embryonic fibroblast
NC: neural crest
CM: conditioned medium
DM: defined medium
GM: growth medium
ESM: embryonic stem medium
ECM: extracellular matrix
Fn: fibronectin
Ln: laminin
